# Stress Doppler echocardiography for early detection of systemic sclerosis-associated pulmonary arterial hypertension

**DOI:** 10.1186/s13075-015-0673-7

**Published:** 2015-06-19

**Authors:** Christian Nagel, Philipp Henn, Nicola Ehlken, Antonello D’Andrea, Norbert Blank, Eduardo Bossone, Anke Böttger, Christoph Fiehn, Christine Fischer, Hanns-Martin Lorenz, Frank Stöckl, Ekkehard Grünig, Benjamin Egenlauf

**Affiliations:** Centre for Pulmonary Hypertension Thoraxclinic, University Hospital Heidelberg, Amalienstr. 5, 69126 Heidelberg, Germany; Lung Centre, Klinikum Mittelbaden, Balger Str. 50, 76532 Baden-Baden Balg, Germany; Department of Cardiothoracic Sciences, Monaldi Hospital, Second University of Naples, Via Leonardo Bianchi, 1, 80131 Naples, Italy; Division of Rheumatology, University Hospital Heidelberg, Im Neuenheimer Feld 410, 69120 Heidelberg, Germany; Department of Cardiology and Cardiac Surgery, University Hospital “Scuola Medica Salernitana”, Via Pr. Amedeo, 36-83023 Lauro (AV) Salerno, Italy; Rheumapraxis Landau, Industriestraße 9, 76829 Landau in der Pfalz, Germany; Department of Rheumatology, ACURA-Klinik Baden-Baden, Rotenbachtalstr. 5, 76530 Baden-Baden, Germany; Department of Human Genetics, University of Heidelberg, Im Neuenheimer Feld 366, 69120 Heidelberg, Germany; Klinikum Darmstadt, Medizinische Klinik III, Grafenstraße 9, 64283 Darmstadt, Germany

## Abstract

**Introduction:**

In patients with systemic sclerosis (SSc), associated pulmonary arterial hypertension (SSc-APAH) is the leading cause of death. The objective of this prospective screening study was to analyse sensitivity and specificity of stress Doppler echocardiography (SDE) in detecting pulmonary hypertension (PH).

**Methods:**

Pulmonary artery pressures and further parameters of PH were assessed by echocardiography and right heart catheterisation (RHC) at rest and during exercise in patients with SSc. Investigators of RHC were blinded to the results of non-invasive measurements.

**Results:**

Of 76 patients with SSc (64 were female and mean age was 58±14 years), 22 (29 %) had manifest PH confirmed by RHC: four had concomitant left heart diseases, three had lung diseases, and 15 had SSc-APAH. Echocardiography at rest missed PH diagnosis in five of 22 patients with PH when a cutoff value for systolic pulmonary arterial pressure (PASP) was more than 40 mm Hg at rest. The sensitivity of echocardiography at rest was 72.7 % (95 % confidence interval (CI) 0.52–0.88), and specificity was 88.2 % (95 % CI 0.78–0.95). When a cutoff value for PASP was more than 45 mm Hg during low-dose exercise, SDE missed PH diagnosis in one of the 22 patients with PH and improved sensitivity to 95.2 % (95 % CI 0.81–1.0) but reduced specificity to 84.9 % (95 % CI 0.74–0.93). Reduction of specificity was partly due to concomitant left heart disease.

**Conclusions:**

The results of this prospective cross-sectional study using RHC as gold standard in all patients showed that SDE markedly improved sensitivity in detecting manifest PH to 95.2 % compared with 72.7 % using echocardiography at rest only. Thus, for PH screening in patients with SSc, echocardiography should be performed at rest and during exercise.

**Trial registration:**

ClinicalTrials.gov NCT01387035. Registered 29 June 2011.

## Introduction

Pulmonary hypertension (PH) is a common complication of systemic sclerosis (SSc) which can occur at any stage of the disease and has been observed in 15–27 % of patients [1, 2]. In most cases, PH is due to pulmonary arterial hypertension (PAH) which is associated with SSc (SSc-APAH). Three-year survival for patients with untreated SSc-APAH has been estimated to be 56 % compared with 91 % in those patients without PAH [3]. According to the french itinerAir study, at PAH diagnosis, more than 80 % of patients with SSc are in World Health Organization (WHO) functional class (FC) II–IV [4]. Today, 10 PAH-targeted drugs are available for these patients [5]. Therefore, an early diagnosis of PH/APAH is essential in patients with SSc.

Echocardiography at rest is the most important non-invasive method for the detection of PH and has been recommended for screening of patients at risk in several guidelines [6–8]. The reliability of tricuspid regurgitation velocity (TRV) cutoff values, with RHC as a reference, has previously been assessed in patients with SSc [9, 10]. A TRV of at least 3.4 m/s with an assumed right atrial (RA) pressure of 5 mm Hg (corresponding to a systolic pulmonary arterial pressure (PASP) of 50 mm Hg) has been recommended as a cutoff value for performing RHC to diagnose or exclude PH in the European Society of Cardiology/European Respiratory Society (ESC/ERS) guidelines [6]. In a multicenter study aiming at the development of a screening algorithm in SSc patients (DETECT study), transthoracic Doppler echocardiography (TDE) at rest using these cutoff values alone was not reliable to detect early forms of SSc-APAH [11]. In this study, RHC was performed in each patient with SSc. The study showed that, of 84 patients with manifest SSc-APAH, only 30 % had a TRV of more than 3.4 m/s at rest and 57 % of more than 2.8 m/s. Thirty-six percent of patients would have been overlooked if only TDE had been used [11]. When using the screening algorithm of the DETECT study, including TDE, diffusing capacity of the lung for carbon monoxide (DLCO), electrocardiogram and further laboratory parameters, only 4 % of patients were missed [11]. Previous studies showed that patients with SSc and increased pulmonary arterial pressure response to exercise were impaired in their physical exercise capacity [12]. Furthermore, they were more prone to develop a manifest PH within 1–3 years [13] and had a worse prognosis [14].

Thus, assessing exercise haemodynamics obtained by RHC and by non-invasive stress Doppler echocardiography (SDE) may identify abnormal pulmonary circulation and may help to identify patients with PH/SSc-APAH at an early stage [15]. However, the role of SDE for PH screening is unclear because of the lack of prospective confirmatory data [6, 7]. Therefore, the objective of this prospective study was to analyse whether SDE improves sensitivity and specificity of detecting PH in comparison with echocardiography at rest. To confirm diagnosis, RHC at rest and during exercise was performed in all patients.

## Methods

### Study population and design

The study was designed as a prospective cross-sectional study in which SSc patients without known PH have been systematically screened by using echocardiography at rest, SDE, and RHC [16]. The investigators who performed echocardiography and SDE were blinded to the results of RHC. To minimise bias, RHC as the confirmatory diagnostic test was performed in each patient.

Patients who had diffuse cutaneous systemic sclerosis (dcSSc) and limited cutaneous systemic sclerosis (lcSSc) (CREST syndrome) and were at least 18 years old were included. Diagnosis of SSc was confirmed by experienced rheumatologists (NB, AB, CFie, H-ML, and FS) according to the standard criteria of the American College of Rheumatology [17]. Exclusion criteria were the following: manifest PH confirmed by RHC prior to enrolment, receiving PH therapy, forced vital capacity (FVC) of less than 40 % of predicted, renal insufficiency, systemic arterial hypertension with pressure values of more than 160/90 mm Hg at rest or more than 220/120 mm Hg during exercise despite optimised medical treatment, previous evidence of clinically relevant left heart disease, or pregnancy.

All patients underwent a detailed clinical work-up, including medical history, physical examination, electrocardiogram, two-dimensional echocardiography at rest and during exercise, lung function test, arterial blood gases, chest x-ray, 6-minute walking distance (6MWD) under standardised conditions [18], laboratory testing including NT-proBNP (N-terminal of the prohormone brain natriuretic peptide) levels, and RHC. Twelve-lead electrocardiogram was performed in all patients (Hellige EK 512 P, Hellige, Freiburg, Germany). Ventilation/perfusion scintigraphy, (Philips Axis, Philips, Hamburg, Germany), computed tomography (Siemens Somatom, Definition AF & Emotion, Siemens, Berlin and Munich, Germany). Scan of the lungs, and left heart catheterisation were performed in all patients with suspected chronic thromboembolic PH, left heart, or respiratory diseases and when clinically indicated. Manifest PH/APAH has been diagnosed according to the current ERS/ESC guidelines [6, 7].

#### Transthoracic Doppler echocardiography at rest

A complete echocardiographic examination was done prior to exercise. Two-dimensional and colour-flow-guided continuous-wave Doppler echocardiographic recordings at rest and during exercise were obtained by experienced cardiac sonographers (EG and CN) using 3.6–4 MHz Duplex probes and conventional equipment (Vivid 7, GE Healthcare, Milwaukee, WI, USA) at rest and during exercise testing as described previously [16]. PASP was estimated from peak tricuspid regurgitation jet velocities according to the equation: PASP = 4 (V)^2^ + RA pressure, where V is the peak velocity (in metres per second) of TRV [19]. For all calculations, the mean value of at least three TRV measurements was used. RA pressure was estimated from characteristics of the inferior vena cava [20]. If it was less than 20 mm in diameter and decreased during inspiration, we added 5 mm Hg; if it was at least 20 mm, we added 10 mm Hg.

#### Stress Doppler echocardiography

Patients were examined on a variable load 45° cycle ergometer (model 8420; KHL Corp., Kirkland, WA, USA) as described previously [16]. Workload was started at 25 Watts and increased by 25 Watts every 2 min to an exercise capacity or symptom-limited maximum. TRV, heart rate, oxygen saturation, and systemic blood pressure were analysed at each stage. Echocardiographic assessment was stored in DICOM (Digital Imaging and Communications in Medicine) format for further offline analysis.

#### Right heart catheter

Patients were examined on a variable load supine bicycle ergometer (model 8420; KHL Corp.) by an experienced investigator (BE) who was blinded to the results of echocardiography and SDE. The examination at rest was performed as previously described [16] in a supine position by using the transjugular access with an 8 F introducer set (MXI100, MEDEX, Smiths Group PLC, London, UK). Catheterisation was done by triple-lumen 7 F-Swan-Ganz thermodilution catheters by Edwards Lifesciences (REF:131 F7, Edwards Lifesciences LLC, Irvine, CA, USA). Pressures were continuously recorded and averaged over several respiratory cycles during spontaneous breathing, both at rest and during exercise. Cardiac output (CO) was measured by thermodilution at least in triplicate with a variation of less than 10 % between the measured values. The zero reference point for pressure recordings was set at 1/3 of the thoracic diameter below the anterior thorax surface [21]. After the hemodynamic measurement at rest, the supine position was changed to a 45° position. The zero reference point was calibrated to the pulmonary arterial wedge pressure (PAWP). After careful calibration, the hemodynamic parameters at rest were measured again and the exercise test was started with a workload of 25 Watts. Workload was incrementally increased by 25 Watts every 2 min to an exercise capacity or symptom-limited maximum. All examinations and measurements were performed by the same experienced team. There were no complications.

#### Cutoff values of systolic pulmonary arterial pressures at rest and during mild to moderate exercise

In this study, we used PASPs of more than 40 mm Hg at rest and of more than 45 mm Hg during low-dose exercise (25–50 Watts over 2 minutes) as cutoff values for the non-invasive detection of manifest PH. These thresholds are based on previous publications, stating that healthy subjects do not exceed these values at rest [16] or during low-dose exercise [22] defined as CO below 10 l/min. Furthermore, with the Chemla formula (mean pulmonary arterial pressure (mPAP) = 0.61*PASP + 2 mm Hg) [23] or the Syyed formula (mPAP = 0.65*PASP +0.55 mm Hg) [24], which both revealed a high accuracy and precision [25], mPAP of 25 mm Hg at rest is equal to a PASP of 38 mm Hg, and mPAP of 30 mm Hg during exercise would reflect a PASP of 45.9 mm Hg. These PASP cutoff values are also within the recommended values mentioned in the ERS/ESC guidelines for PH [16].

### Ethics statement

This study was conducted in accordance with Good Clinical Practice and the current version of the revised Declaration of Helsinki (World Medical Association Declaration of Helsinki). The ethics committee of the University of Heidelberg approved the study (Internal Ethics-Nr. S-360/2009). Written informed consent was obtained from each patient prior to enrolment.

### Statistical methods

Baseline was defined as the day when the patient underwent echocardiography at rest and SDE. PH and non-PH groups were described by using summary statistics: sample size, mean, and standard deviation for quantitative data and frequencies (counts and percentages) for qualitative and categorical data. Differences between groups were analysed with an unpaired two-sided Student’s *t* test. All tests were two-sided, and *P* values of less than 0.05 were considered statistically significant.

Sensitivity and specificity for echocardiography at rest and during exercise using cutoff values for PASP of 40 mm Hg at rest and 45 mm Hg during exercise were calculated separately and in combination of the two methods compared with RHC as gold standard. We report 95 % confidence intervals (CIs) for sensitivity and specificity estimates. For the combination of echocardiography at rest and during exercise, a patient was classified as having PH when being above the threshold for at least one method. For further analysis of suitability of the thresholds, receiver operating characteristic (ROC) and area under the curve estimates with 95 % confidence intervals are shown. We also performed multiple regression analysis with stepwise forward selection of parameters for predicting mPAP at rest as metric variable and also for binary classification of PH (mPAP ≥25 mm Hg versus mPAP < 25 mm Hg). Parameters included in multiple regression were age, gender, DLCO (percentage), FVC (percentage), systolic pulmonary arterial pressure at rest, at 25 Watts, RA area, right ventricular (RV) area, and tricuspid annular plane systolic excursion (TAPSE). All analyses were carried out with IBM SPSS 20 (SPSS Statistics version 20, IBM Corporation, Armonk, NY, USA).

## Results

### Study population

During the study, 81 patients with SSc were screened, and five were excluded for the following reasons: the diagnosis of SSc was not confirmed in three patients by an experienced rheumatologist, and two patients were not willing to perform an RHC. Thus, the final study group consisted of 76 consecutive patients with SSc: 59 % (*n* = 45) of these patients were diagnosed with dcSSc, and 41 % (*n* = 31) with lcSSc. The mean age was 58±14 years, and there were 12 (16 %) males and 64 (84 %) females (Table 1). The youngest patient was 28 years old; the oldest was 82 years old. The duration of SSc at the time of the first visit was 12.0±11.3 years, and the minimum duration was 16 months. None of these patients had a history of RHC or targeted oral medication for PH or digital ulcerations. Clinical findings at baseline are shown in Table 1.

**Table 1:** Characteristics of scleroderma patients

Number of included patients		76	
Gender, female/male	64	/	12
Age, years	57.9	±	14.4
Duration of systemic sclerosis, years	12.0	±	11.3
Body weight, kg	68.8	±	14.0
Body height, cm	165.5	±	8.1
Body surface area, m^2^	1.8	±	0.2
WHO functional class			
0-I	17		22 %
II	31		41 %
III	27		36 %
6-minute-walking-distance, m	435	±	94.4
Borg Dyspnoea Scale (6-20)	13.8	±	2.4
Haemodynamics by right heart catheter			
mPAP, mm Hg	20.1	±	9.6
PVR, dynes*sec per cm^5^	181.4	±	151.9
PAWP, mm Hg	8.8	±	4.9
Cardiac output, l/min	5.4	±	1.2
Cardiac index, l/min per m^2^	3.0	±	0.6

Values are given as number, mean±standard deviation, or number and percentage

*WHO* World Health Organization, *mPAP* mean pulmonary arterial pressure, *PVR* pulmonary vascular resistance, *PAWP* pulmonary arterial wedge pressure

At baseline, 17 of the 76 patients (22 %) presented with no signs of dyspnoea in everyday life and during exercise and therefore were categorized as WHO FC 0-I. Thirty patients (41 %) were categorized as WHO FC II, and 27 patients (36 %) as WHO FC III. WHO FC was not documented in one patient.

### Diagnosis of manifest pulmonary hypertension by right heart catheterisation

**Fig. 1:**
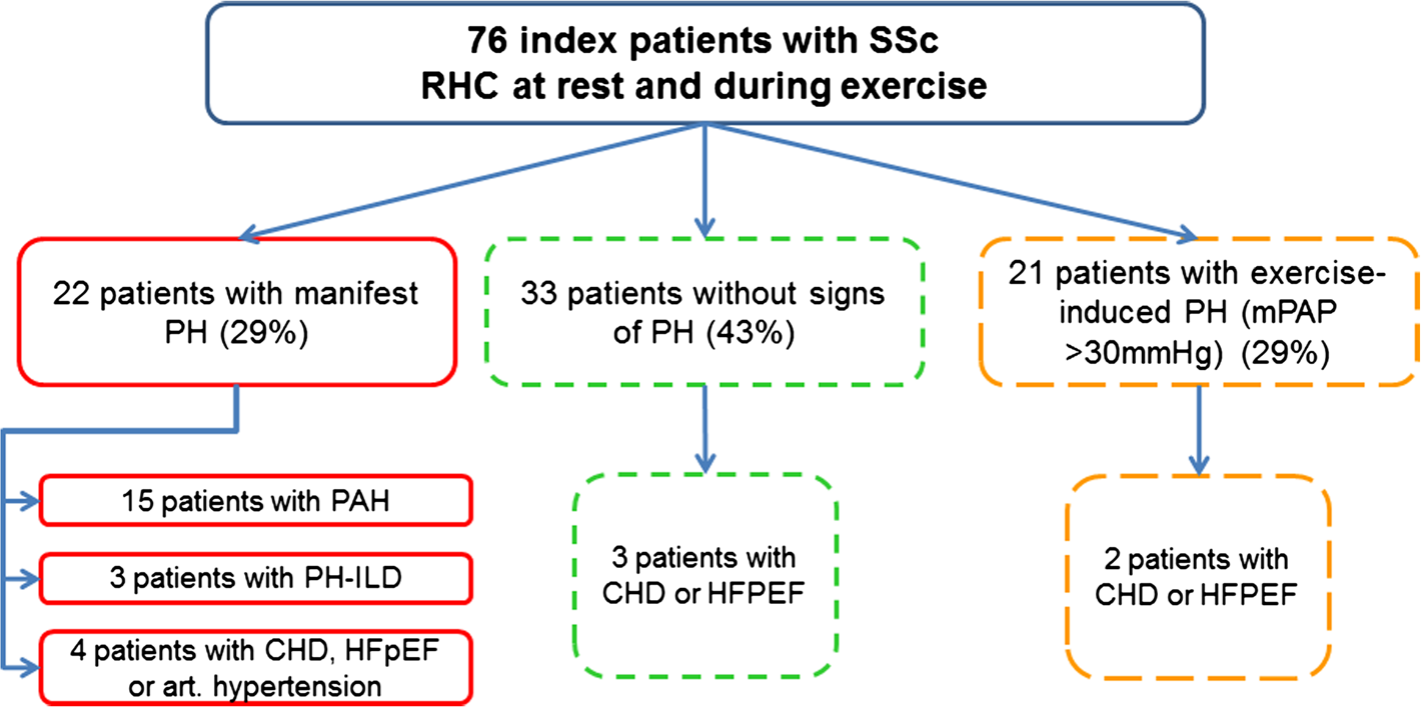
Results of the screening of 76 patients with systemic sclerosis (SSc) by right heart catheterisation (RHC). This figure shows that within the screening assessment 29 % of the 76 patients were newly diagnosed with a manifest pulmonary hypertension (PH) and 43 % had no signs of PH. *CHD* coronary heart disease, *HFpEF* heart failure with preserved ejection fraction, *mPAP* mean pulmonary arterial pressure, *PH-ILD* pulmonary hypertension-interstitial lung disease

Twenty-two (29 %) of 76 patients with SSc had an elevated mPAP of at least 25 mm Hg and therefore a manifest PH (Fig. 1). Four of these 22 patients with PH showed an elevated PAWP of more than 15 mm Hg. Further diagnostic work-up by left heart catheter revealed a coronary heart disease or left heart failure with preserved ejection fraction (HFpEF) (or both) in two patients. Signs of significant pulmonary fibrosis in a CT scan and an FVC of less than 70 % were found in four patients with PH. Three patients were classified as having PH-interstitial lung disease. One patient presented with a PAWP of more than 15 mm Hg and was classified as having PH with left heart disease after further diagnostic work-up, including left heart catheterisation.

In 21 patients (28 %), mPAPs were between 21 and 24 mm Hg at rest (borderline) or more than 30 mm Hg during exercise or both. In two patients out of this group, coronary heart disease or HFPEF or both were diagnosed by left heart catheterisation (Fig. 1). Thirty-three patients with SSc (43 %) presented with normal mPAP at rest and during exercise (Fig. 1).

Patients with newly diagnosed manifest PH revealed a mean mPAP of 33±8 mm Hg and a mean pulmonary vascular resistance of 339±199 dynes*s per cm^5^, indicating an early diagnosis reached by the screening procedure of this study (Table 2).

**Table 2:** Comparison between no pulmonary hypertension and manifest pulmonary hypertension group

Diagnosis		No PH		Manifest PH		
		mPAP < 25 mm Hg		mPAP ≥ 25 mm Hg		
		*n* = 54		*n* = 22		
		Mean	SD	Mean	SD	*P* value
Patient characteristics	Age, years	54.0	14.4	67.6	8.8	<0.001
	Height, cm	167.3	7.8	162.1	8.1	0.02
	Weight, kg	70.0	16.0	66.0	10.5	n.s.
	Body surface area, m^2^	1.77	0.2	1.7	0.2	n.s.
	Duration of SSc, years	10.7	9.9	15.6	14.4	n.s.
	Beginning of SSc, years	44.5	15.0	52.4	16.0	0.04
	Modified Rodnan Skin Score	13.3	8.5	18.4	11.4	0.04
	6MWD, m	466	77	349	86	<0.001
	Borg Dyspnoea 20	13	2	15	2	0.003
WHO FC	0-I	17 (31.5 %)		0		
	II	26 (48.1 %)		5 (22.7 %)		
	III	10 (18.5 %)		17 (77.3 %)		
	Unknown	1 (1.9 %)		0		
Laboratory	NT-proBNP, pg/mL	290	424	846	773.0	0.02
Blood gas analysis	Oxygen saturation, %	96.8	2.1	94.6	3.4	0.01
Lung function	Vital capacity, %	95.8	24.2	88.2	29.9	n.s.
	FEV1, %	108.0	93.7	83.7	26.9	n.s.
	Total lung capacity, %	95.5	21.4	83.8	23.2	0.042
	Residual volume, %	2.0	0.8	1.9	0.6	n.s.
Diffusion capacity	Diffusion capacity carbon monoxide, %	55.7	13.5	42.1	12.5	<0.001
Echocardio-	RV thickness, mm	6.5	1.2	7.6	1.4	0.01
graphy	TAPSE, mm	24.0	3.5	20.6	4.0	0.001
	RA area, cm^2^	11.4	3.3	15.5	5.7	<0.001
	RV area, cm^2^	14.6	3.7	17.7	4.2	0.003
	TRV, m/s	2.3	0.4	3.4	0.6	<0.001
	PASP, mm Hg	25.6	7.3	52.0	18.0	<0.001
	PASP max (exercise), mm Hg	49.9	12.7	83.9	18.9	<0.001
Right heart	RAP/CVP, mm Hg	3.8	2.6	6.3	4.5	0.02
catheterisation	mPAP, mm Hg	14.8	3.4	32.6	7.5	<0.001
at rest	PAWP, mm Hg	7.2	3.2	12.4	6.0	0.001
	Cardiac output, ml/min	5.6	1.3	5.1	1.0	n.s.
	Cardiac index, l/min per m^2^	3.1	0.6	2.9	0.5	n.s.
	PVR, dynes*s/cm^5^	114	40	339	199	<0.001
During exercise	mPAP max, mm Hg	31	7	50	7	<0.001
	PAWP max, mm Hg	17	6	21	9	n.s.
	Cardiac output max, l/min	11.5	3.4	8.3	2.9	<0.001
	PVR max, dynes*s/cm^5^	96	42	323	207	<0.001

*PH* pulmonary hypertension, *mPAP* mean pulmonary arterial pressure, *SD* standard deviation, *SSc* systemic sclerosis, *6MWD* 6-minute walking distance, *WHO FC* World Health Organization functional class, *NT-proBNP* N-terminal of the prohormone brain natriuretic peptide, *n.s.* not significant, *FEV1* forced expiratory volume in 1 second, *RV* right ventricle, *TAPSE* tricuspid annular plane systolic excursion, *RA* right atrium, *TRV* tricuspid regurgitation velocity, *PASP* systolic pulmonary arterial pressure, *RAP* right atrial pressure, *CVP* central venous pressure, *PAWP* pulmonary arterial wedge pressure, *PVR* pulmonary vascular resistance

*P* values are based on two-sided, unpaired Student’s *t* tests and Mann-Whitney *U* test for Borg Dyspnoea Scale score. For Borg Dyspnoea Scale score, median values equal means. Values are mean±SD. For the category of NT-proBNP, values were missing for 14 patients in the mPAP < 25 mm Hg and eight patients in the mPAP ≥ 25 mm Hg group. For the category of RV thickness, values were missing for seven patients for each group. For the categories of RA area, RV area, and TRV, values for one patient were missing in the mPAP < 25 mm Hg group. For the category of PASP, values were missing for three patients in the mPAP < 25 mm Hg group. For the category of PASP max (exercise), values were missing for three patients in the mPAP < 25 mm Hg group. For the categories of cardiac index, mPAP max, PWAP max, cardiac output max, and cardiac index max, values for one patient was missing for each group, respectively

### Comparison between patients without and with pulmonary hypertension

Patients in the PH group were significantly older with a later SSc diagnosis and had a higher Modified Rodnan Skin Score and therefore a more severe skin involvement (Table 2). Patients with PH had been categorized in higher WHO FC and revealed significantly lower 6MWD and a higher Borg Index and NT-proBNP levels than patients without PH. Patients with PH had a significantly larger RA and RV area, larger thickness of the free RV wall, and a lower tricuspid annular plane systolic excursion than non-PH patients (Table 2). There were no significant differences between the two groups concerning CO at rest. During maximum exercise, the PH group showed a significantly lower CO than the non-PH group and had a worse RV contractile reserve (Table 2).

### Sensitivity and specificity of echocardiography at rest versus stress Doppler echocardiography during exercise

**Fig. 2:**
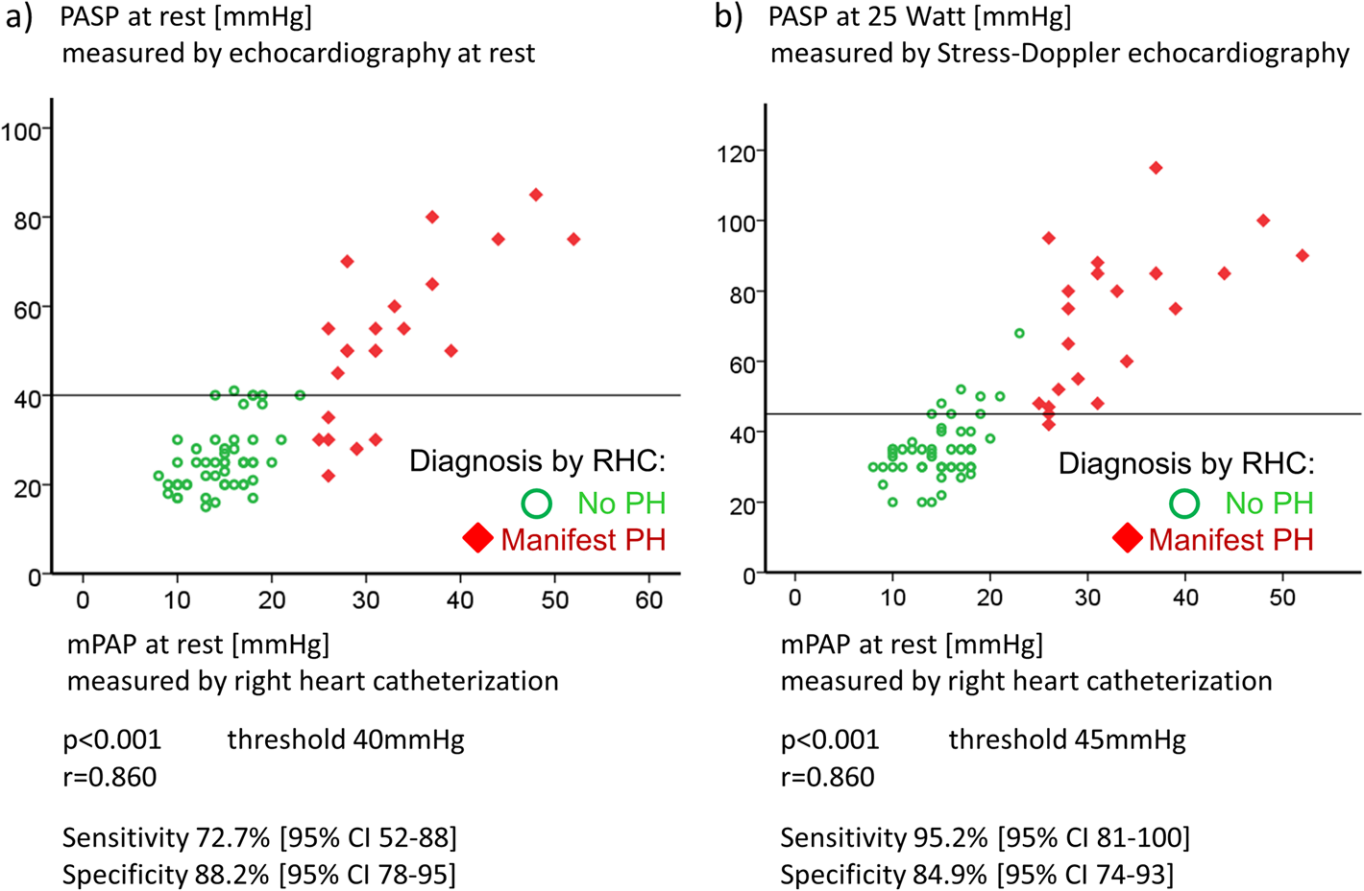
**a** Correlation of systolic pulmonary arterial pressures (PASP) determined by echocardiography and mean pulmonary arterial pressure (mPAP) at rest. The x-axis shows the PASP values measured by echocardiography at rest, the y-axis the values measured by right heart catheterisation (RHC). The values of each patient are given in red, indicating that the assessment including RHC diagnosed a manifest pulmonary hypertension (PH) with an mPAP at rest of at least 25 mm Hg. The symbols in green are the values of systemic sclerosis patients with no manifest PH at rest. As cutoff value for the PASP, 40 mm Hg at rest was used. The sensitivity was 72.7 % (95 % confidence interval (CI) 52–88 %) and the specificity was 88.2 % (95 % CI 78–95 %) in diagnosing a PH (*P* < 0.001). There was a positive correlation (r = 0.860) between PASP and mPAP. **b** Correlation of PASP at 25 Watts and mPAP at rest. The x-axis shows the PASP values measured by echocardiography during low-dose exercise at 25 Watts. The y-axis shows the values measured by RHC at rest. Sensitivity was 95.2 % (95 % CI 81–100 %) and specificity was 84.9 % (95 % CI 74–93 %) in diagnosing a PH using a cutoff PASP value of 45 mm Hg at 25 Watts (*P* < 0.001). Positive correlation (r = 0.860) between PASP at 25 Watts and mPAP

Echocardiography at rest revealed a sensitivity in diagnosing manifest PH of 72.7 % (95 % CI 52–88 %) and a specificity of 88.2 % (95 % CI 78–95 %) when a cutoff PASP value of 40 mm Hg was used (Fig. 2a).

SDE with a workload of 25 Watts was possible in 74 (97 %) of the 76 patients. One patient refused exercise testing; another patient had to miss the exam because of a foot injury. A further eight patients had to stop exercise testing before cardiopulmonary limitation because of orthopaedic problems: six patients (7.9 %) had coxarthrosis or knee arthrosis or both, and two patients (2.6 %) had knee replacement implants. However, these patients completed the workload of 25 Watts and could be included in the exercise echocardiography analysis.

SDE had a sensitivity in diagnosing manifest PH by measuring the PASP during low-dose exercise (25 Watt) of 95.2 % (95 % CI 81–100 %) and a specificity of 84.9 % (95 % CI 74–93 %) when a cutoff value of more than 45 mm Hg was used (Fig. 2b).

In multiple regression analysis, at 25 Watts was the most important independent parameter to determine mPAP (R = 0.856, *P* > 0.001). For binary classification of diagnosis of PH, at 25 Watts was the only independent predictor (R = 0.779, *P* < 0.001).

**Fig. 3:**
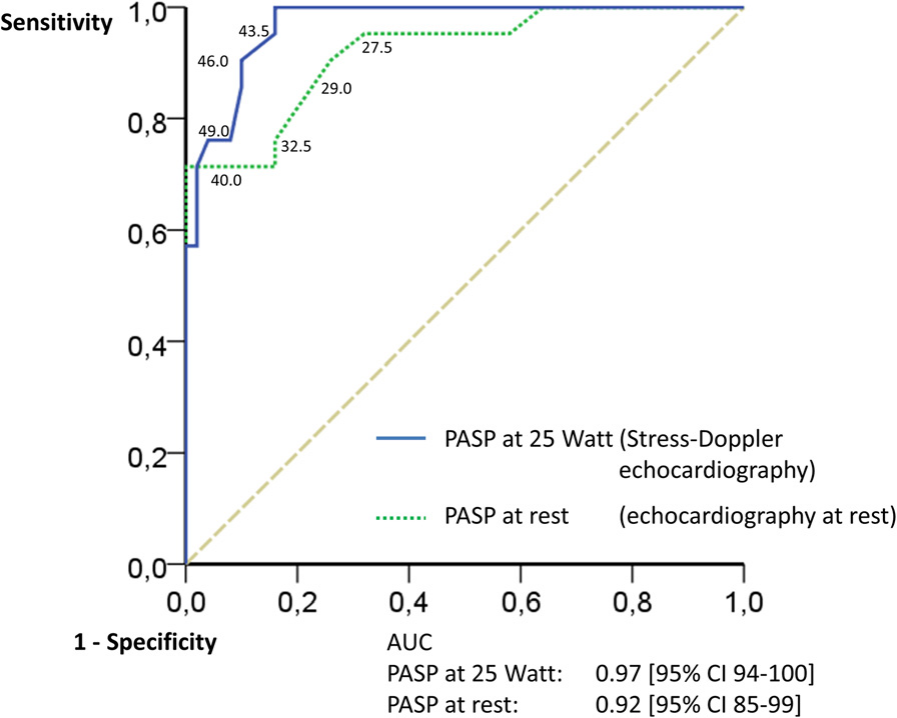
Receiver operating characteristic (ROC) curves of sensitivity and specificity of systolic pulmonary arterial pressures (PASP) at rest and at 25 Watts. The threshold for detection of pulmonary hypertension was set at 40 mm Hg for echocardiography at rest and at 45 mm Hg for echocardiography during exercise. All possible thresholds were analysed by ROC analysis for their suitability. The cutoff value for echocardiography during exercise at 45 mm Hg revealed the highest combination of sensitivity and specificity. For the examination at rest, a decrease of the cutoff value would have led to an increase of sensitivity but a crucial decrease of specificity. *AUC* area under the curve, *CI* confidence interval

The two thresholds, 40 mm Hg PASP at rest and 45 mm Hg PASP during exercise at 25 Watts, were analysed by ROC analysis for their suitability (Fig. 3). The cutoff value for echocardiography during exercise at 45 mm Hg revealed the highest combination of sensitivity and specificity. For the examination at rest, a lower cutoff value would have led to an increase of sensitivity but a crucial decrease of specificity.

**Fig. 4:**
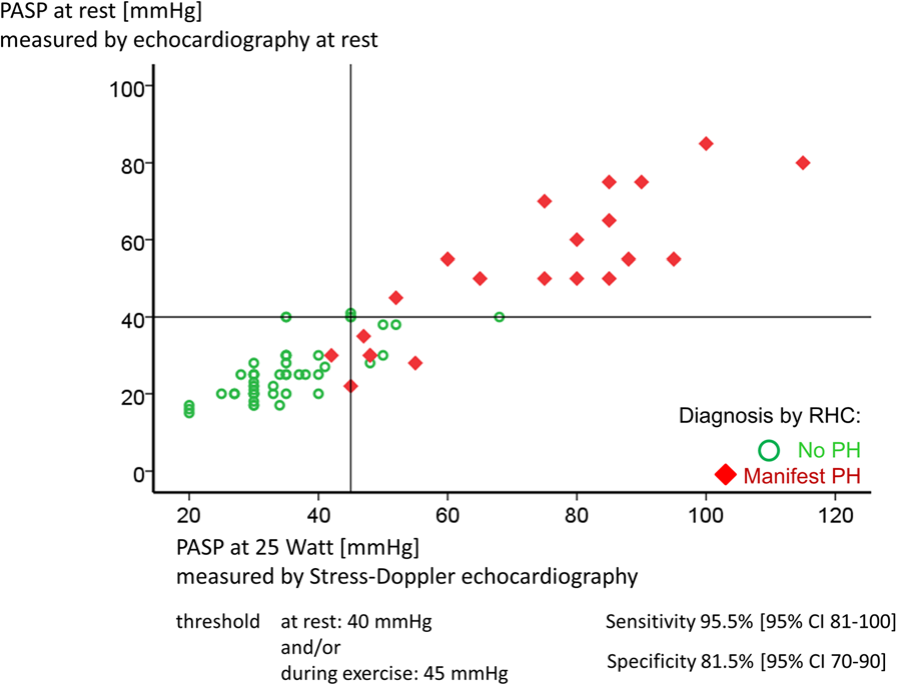
Correlation of systolic pulmonary arterial pressure (PASP) at 25 Watts and PASP at rest. Cutoff values are 45 mm Hg for PASP at 25 Watts and 40 mm Hg PASP at rest. Circles represent patients without pulmonary hypertension (PH) verified by right heart catheterisation (RHC); squares are patients with manifest associated pulmonary arterial hypertension according to RHC as gold standard. PASP at rest with a cutoff value of 40 mm Hg would have missed five manifest PH patients; PASP at 25 watts with a cutoff value of 45 mm Hg would have missed only one patient with a slightly lower specificity. *CI* confidence interval

It was even possible to increase the sensitivity of diagnosing a manifest PH by combination of TDE at rest and during exercise, as shown in Fig. 4 (the squares symbolize those patients with an invasively diagnosed manifest PH). With cutoff values of 40 mm Hg for PASP at rest and of 45 mm Hg at a workload of 25 Watts, 21 out of 22 patients with manifest PH have been identified. A comparison of different screening algorithms including the combination of PASP at rest and at 25 Watts is given in Table 3.

**Table 3:** Comparison of screening algorithms for pulmonary hypertension in systemic sclerosis

Approach	False negatives, % (missed diagnoses)	Sensitivity, %	Specificity, %	PPV, %	NPV, %
SDE at rest and during exercise	1	96	82	68	98
*N* = 76					
DETECT algorithm	4	96	48	35	98
*N* = 319					
DETECT algorithm with	15	85	72	47	94
65 % specificity at step 2					
*N* = 319					
DETECT data with algorithm from					
ESC/ERS guidelines*	29	71	69	40	89
*N* = 371					

*PPV* positive predictive value (confirmed pulmonary arterial hypertension out of all right heart catheterization referrals), *NPV* negative predictive value, *SDE* stress Doppler echocardiography, *ESC/ERS* European Society of Cardiology/European Respiratory Society

Adaptation of the table provided by Coghlan et al. [11] in the DETECT study

*Evaluated on a subset of patients from DETECT study (*n* = 371) with available data for the variables defined in the guideline

Criteria were the following: (a) tricuspid regurgitant jet velocity >3.4 m/s or (b) tricuspid regurgitant jet velocity >2.8 to ≤ 3.4 m/s AND symptomatic (defined as at least one of the following DETECT parameters: current anginal pain, current syncope/near syncope, current dyspnoea, or presence of peripheral oedema) or (c) tricuspid regurgitant jet velocity ≤2.8 m/s AND symptomatic (defined as above) AND presence of additional echocardiography variables suggestive of pulmonary hypertension (defined as right atrium area >16 cm^2^ or ratio of right ventricular diameter/left ventricular end diastolic diameter >0.8 or both)

One patient who would have been missed by the combination of TDE at rest and SDE presented with only a mild PH with an mPAP of just 26 mm Hg and PASP of less than 45 mm Hg at rest and during exercise.

Six further patients had PASPs at 25 Watts of more than 45 mm Hg with an mPAP of less than 25 mm Hg. These patients were “false-positive” because of elevated PAWP during exercise. The further diagnostic work-up showed that two of them had coronary heart disease and that four had an HFpEF and therefore a post capillary exercise-induced PH and no PAH.

### Safety

In the examination by SDE in all patients, TRV could be successfully measured at rest and during exercise because of the presence of tricuspid regurgitation in the enlarged right hearts. SDE and RHC during low workloads were safe and could be performed without complications in all patients.

## Discussion

This is the first prospective study evaluating sensitivity and specificity of echocardiography at rest and during low-dose exercise (25 Watts, CO <10 l/min) compared with RHC in patients with SSc. With cutoff values of PASP of 40 mm Hg at rest and 45 mm Hg during low-dose exercise, SDE markedly improved the sensitivity to identify patients with manifest PH (diagnosed by RHC) from 72.7 % to 95.2 % compared with echocardiography at rest only. Specificity of PAH diagnosis by SDE was 84.9 % and was reduced mainly because of concomitant cardiovascular diseases. SDE detected even patients with only mild manifest PH/PAH-SSc at an early stage (mPAP of 25–30 mm Hg), which was often overlook by echocardiography at rest only. Thus, SDE may be a useful non-invasive technique to identify SSc patients with PH/PAH at an early stage.

### Pulmonary hypertension screening in systemic sclerosis patients using transthoracic Doppler echocardiography at rest

Echocardiography at rest has been used for PH screening in SSc patients for more than 20 years [26]. However, in contrast to our study, all previous studies except the DETECT study did not systematically use RHC in all patients. Denton et al. [27] found a higher sensitivity of 90 % and a specificity of 75 % for echocardiography at rest and identified manifest SSc-APAH by RHC in 64 % of patients. However, this was a highly pre-selected patient cohort. In our prospective study, patients have been consecutively included even when they had no symptoms. Mukerjee et al. [9] described sensitivity and specificity values according to different cutoff values of PASP at rest of at least 30 mm Hg, at least 35 mm Hg, at least 40 mm Hg, and at least 45 mm Hg of 88/42 %, 75/66 %, 58/87 %, and 47/97 %, respectively. Their conclusion was that TDE at rest is not a useful screening tool for early SSc-APAH but may provide a moderate specificity in advanced PAH. Hsu et al. detected a sensitivity of TDE diagnosing PH in a high-risk cohort of patients with symptomatic SSc (49 % were diagnosed with PH) of 58 % using a cutoff level of 47 mm Hg [28]. These previous findings are in contrast to those of the DETECT study, in which RHC was performed in each patient with SSc. Of the 85 patients with manifest SSc-APAH, only 29.8 % had a TRV of at least 3.4 m/s at rest corresponding to a cutoff PASP value of 50 mm Hg [11]. Thirty-six percent of patients with manifest PH would have been overlooked using TDE at rest only [11] using a cutoff value of 2.8 m/s corresponding to 40 mm Hg at rest. The results of the DETECT study have been confirmed in a retrospective analysis showing that a threshold of more than 3.4 m/s for TRV would have been missed in 48 % of patients [29]. The sensitivity of TDE at rest in our study was higher and this might be because the DETECT study presented a “real-world” echocardiographic assessment without clear pre-evaluation of investigators and training of this method. In contrast, in our study, TDE was more standardised. The results of our study suggest that accuracy of TDE could probably increase even more if further parameters such as RA and ventricular area were included. This was also recommended in the Delphi consensus study [30]. The current recommendation from the Scleroderma Foundation and Pulmonary Hypertension Association for screening of PH [31] is to use a combination of DLCO, TDE (both high evidence), NT-proBNP, and the DETECT algorithm if DLCO% is less than 60 % and disease duration is more than 3 years (both moderate evidence) [11].

### Role of stress Doppler echocardiography in the screening for pulmonary hypertension in patients with systemic sclerosis

Within the last 10 years, SDE has been increasingly used to assess pulmonary circulation [15]. Alkotob et al. (2006) reported for the first time that a PASP increase more than 40 mm Hg during exercise was associated with a higher risk of developing manifest PH [13]. Furthermore, patients with exercise-induced PH reached lower workloads [12] and exercise time [13]. Steen et al. (2008) identified 17 out of 21 patients with ΔPASP increase of more than 20 mm Hg during exercise and suggested that these patients are at risk for PH [32]. All of these patients were symptomatic, but only 19 % of patients had a manifest PH according to the current definition (mPAP of at least 25 mm Hg). Kovacs et al. (2010) showed a close correlation between pulmonary artery pressures (PAPs) obtained by SDE and RHC during exercise and confirmed less 6MWD in patients with SSc-APAH [12]. Codullo et al. (2013) demonstrated, in a low-risk cohort of 170 patients, that increased ∆PASP during SDE is a risk factor for the development of manifest PH [33]. All of these studies showed certain aspects of SDE and identified elevated PASP during exercise as a risk factor for the development of pulmonary vascular disease. However, none of these studies assessed sensitivity and specificity of this method in identifying PH using RHC as the gold standard in all patients. Our results indicate that SDE can be a reliable non-invasive screening method for early manifest PAH in SSc if the assessment is standardised. There are the first data indicating that an early treatment with PH-targeted therapy might attenuate PASP increase in patients with borderline PH in SSc [34]. However, the role of early treatment in SSc-APAH is still to be investigated by randomised controlled trials.

### Which cutoff value for systolic pulmonary arterial pressures is useful?

The current ESC/ERS guidelines did not recommend SDE [6] and this was due mainly to the lack of agreement on cutoff values for PASP during exercise. A PASP of 40 mm Hg is usually taken as the upper limit of normal [4, 35–37], even though this value may be exceeded during exercise by athletes with a CO far above 10 l/min [38]. Therefore, assessment of exercise-induced increases in PAPs should be interpreted relative to increases in blood flow (i.e., ΔPASP/ΔCO) and specific work rates rather than relying on a single absolute PASP or mPAP threshold or a peak exercise PAP [15, 39, 40]. Therefore, in this study, we used PASPs of 40 mm Hg at rest and of 45 mm Hg during low-dose exercise (25 Watts) with COs clearly less than 10 l/min as cutoff values. This is in line with previous data in healthy subjects [16, 38] and methodological prediction formulas (Syyed and Chemla) of PAP [23–25].

### Limitations

Results of SDE can be affected by concomitant diseases such as left heart disease or lung diseases. In our study, the presence of left ventricular dysfunction associated with high PAWP led to false-positive results in SDE [41, 42]. Therefore, in all patients with elevated PAP and high systemic blood pressures or pulmonary artery wedge pressures, a left heart catheterisation has been performed.

Furthermore, in any screening program, it cannot be excluded that there is a referral bias due to the fact that predominantly those patients are willing to perform the screening who already feel some symptoms. Nevertheless, we have included consecutive patients despite symptoms which resulted in a high proportion of patients (22 %) with no or only mild symptoms in our cohort.

TDE and SDE are somewhat dependent on experienced investigators and standardisation of the procedure. Thus, these results may not present a non-standardised screening process. In general, standardisation might be necessary and useful when screening SSc patients for PH. The underlying disease, especially in the case of dcSSc with interfering musculoskeletal involvement, may have influenced the results of the exercise test. The parameter of PASP during exercise in this study corresponds to a workload of 25 Watts. An impaired exercise capacity is therefore assumed to have no crucial impact on the results.

## Conclusions

The results of this prospective cross-sectional study using RHC as gold standard in all patients showed that SDE markedly improved sensitivity in detecting manifest PH to 95.2 % compared with 72.7 % in echocardiography at rest in patients with SSc. Thus, for PH screening in patients with SSc, it might be useful to perform echocardiography at rest and during exercise.

## Abbreviations

6MWD: 6-minute walking distance
APAH: Associated pulmonary arterial hypertension
CI: Confidence interval
CO: Cardiac output
CT: Computed tomography
dcSSc: Diffuse cutaneous systemic sclerosis
DLCO: Diffusing capacity of the lung for carbon monoxide
ERS: European Respiratory Society
ESC: European Society of Cardiology
FC: Functional class
FVC: Forced vital capacity
HFpEF: Heart failure with preserved ejection fraction
lcSSc: Limited cutaneous systemic sclerosis
mPAP: Mean pulmonary arterial pressure
NT-proBNP: N-type bro brain natriuretic peptide
PAH: Pulmonary arterial hypertension
PAP: Pulmonary artery pressure
PASP: Systolic pulmonary arterial pressure
PAWP: Pulmonary arterial wedge pressure
PH: Pulmonary hypertension
RA: Right atrial
RHC: Right heart catheterisation
ROC: Receiver operating characteristic
RV: Right ventricular
SDE: Stress Doppler echocardiography
sPAP: Systolic pulmonary arterial pressure
SSc: Systemic sclerosis
SSc-APAH: Systemic sclerosis associated pulmonary arterial hypertension
TDE: Transthoracic Doppler echocardiography
TRV: Tricuspid regurgitation velocity
WHO: World Health Organization
